# Autophagy in Stem Cell Biology: A Perspective on Stem Cell Self-Renewal and Differentiation

**DOI:** 10.1155/2018/9131397

**Published:** 2018-01-21

**Authors:** Xihang Chen, Yunfan He, Feng Lu

**Affiliations:** Department of Plastic and Cosmetic Surgery, Nanfang Hospital, Southern Medical University, 1838 Guangzhou North Road, Guangzhou, Guangdong 510515, China

## Abstract

Autophagy is a highly conserved cellular process that degrades modified, surplus, or harmful cytoplasmic components by sequestering them in autophagosomes which then fuses with the lysosome for degradation. As a major intracellular degradation and recycling pathway, autophagy is crucial for maintaining cellular homeostasis, as well as for remodeling during normal development. Impairment of this process has been implicated in various diseases, in the pathogenic response to bacterial and viral infections, and in aging. Pluripotent stem cells, with their ability to self-replicate and to give rise to any specialized cell type, are very valuable resources for cell-based medical therapies and open a number of promising avenues for studying human development and disease. It has been suggested that autophagy is vital for the maintenance of cellular homeostasis in stem cells, and subsequently more in-depth knowledge about the regulation of autophagy in stem cell biology has been acquired recently. In this review, we describe the most significant advances in the understanding of autophagy regulation in hematopoietic and mesenchymal stem cells, as well as in induced pluripotent stem cells. In particular, we highlight the roles of various autophagy activities in the regulation of self-renewal and differentiation of these stem cells.

## 1. Introduction

Autophagy, meaning “self-eating” in Greek, is defined as a cellular process responsible for the degradation of cytosolic proteins and subcellular organelles in lysosomes [1]. This process occurs at a basal level in most tissues, contributing to the routine turnover of cytoplasmic components, and as part of tissue homeostasis. Generally, autophagy can be induced by starvation or other forms of cellular stress, which results in lysosomal degradation and recycling of the resulting degradation products to generate cellular building blocks and energy for cellular renovation and homeostasis [2]. Beside this important recycling function, autophagy is increasingly recognized as a quality control mechanism for both proteins and organelles [3–5]. Induced by energy or nutrient starvation or a quality control mechanism, autophagy regulates a number of essential cellular processes including self-renewal, differentiation, senescence, and apoptosis [6–8]. Three types of autophagy are generally considered to occur in mammals: macroautophagy [9], microautophagy [10], and chaperone-mediated autophagy [11]. Macroautophagy is the major type of autophagy observed in most cells, and consequently, it has been the most extensively studied compared to the other types; hence, for the purposes of this review, we will refer to macroautophagy as “autophagy.”

**Figure 1:**
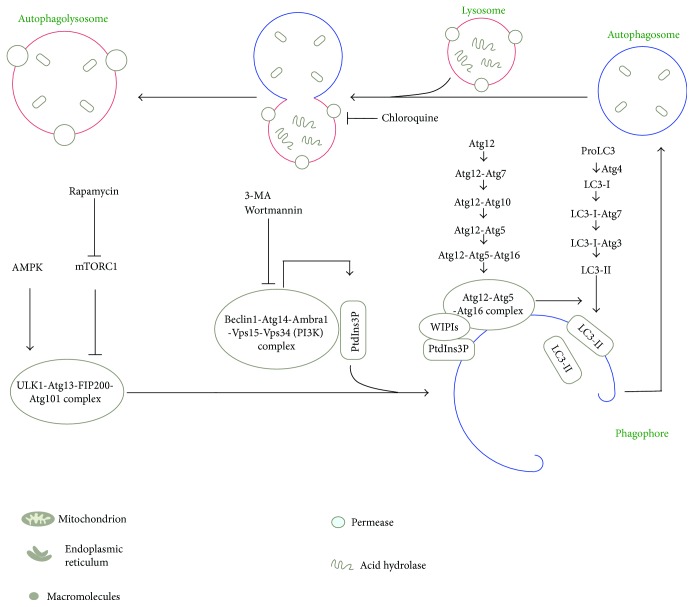
Schematic depiction of the autophagy pathway and potential targets for modulating autophagy. mTORC1 activity suppression or AMPK activation leads to the activation of the ULK1 complex, formed by ULK1, ATG13, FIP200, and ATG101. The active ULK1 complex and the class III phosphatidylinositol-3-phosphate (PtdIns3P) kinase complex, formed by BECN1, ATG14, VPS15, VPS34, and Ambra1, control the initiation of autophagosome, via PtdIns3P formation and WIPI recruitment. The Atg-Atg12-Atg16 complex and LC3-II control the formation of autophagosome. Autophagy can be activated by drugs such as rapamycin that induce autophagy through mTOR inhibition. In contrast, inhibition of class III PI3K by 3-MA can inhibit autophagy. In addition, chloroquine inhibits lysosomal enzymes and also prevents the fusion of autophagosome and lysosome, resulting in the inhibition of autophagy.

Autophagy can be thought of as a process of cellular self-cannibalism in which cytoplasmic components (i.e., macromolecules [12] and organelles [13, 14]) are sequestered and enclosed within double- or multimembraned vesicles (autophagosomes), which then fuses with the lysosome to become an autolysosome and degrade the materials contained within it. Hydrolytic enzymes in the lysosome degrade the content of the autophagosome, and the resulting breakdown products, such as amino acids and fatty acids, are then recycled [15] (Figure 1). The formation of the autophagosome is tightly controlled by the sequential activation of a series of well-characterized protein complexes. For example, the ULK1–ATG13–FIP200–ATG101 complex is responsible for the induction of autophagy [16, 17], the class III phosphatidylinositol (PtdIns) 3-kinase complex (BECN1, ATG14/ATG14L, VPS15, VPS34, and AMBRA1) is responsible for the initiation of the autophagosome [18, 19], and the ATG12-5-16 and LC3-II are responsible for the formation of autophagosome [20–22]. Specifically, Atg12 is a ubiquitin-like protein that is activated at its C-terminus by the E1 enzyme Atg7 and then transferred to the E2 enzyme Atg10 before being covalently linked to Atg5 [23]. This Atg12-Atg5 conjugate, together with Atg16, forms a complex (Atg12/Atg5/Atg16) that is essential for autophagy [24]; this system is also conserved in mammalian cells [25]. A second system utilizes enzymatic cleavage of the precursor Atg8 by Atg4, with the resultant cleaved Atg8 being covalently bound to the lipid phosphatidylethanolamine (PE) through an amide bond by the sequential actions of the E1 enzyme Atg7 and the E2 enzyme Atg3; this latter process is facilitated by the Atg12/Atg5/Atg16 complex referred to above [26]. Upon autophagosome maturation and fusion of its outer membrane with the lysosome membrane, the autophagosome contents, as well as its inner membrane, are degraded to generate amino acids and other cellular building blocks for recycling by the cell.

Autophagy is a highly conserved process that is regulated by complex signaling pathways. Among these signaling pathways, the mammalian target of rapamycin (mTOR) and AMP-activated protein kinase (AMPK) pathways are the two major pathways that regulate autophagy in mammals [27]. In response to nutrient-rich, low cell stress conditions, the mTOR pathway is activated and promotes protein translation and cell growth. Activation of the mTOR pathway by specific depletion of tuberous sclerosis complex 1 (TSC1) inhibits autophagy [28, 29]. In contrast, activation of the AMPK pathway induces autophagy. Under conditions of metabolic stress, the AMPK pathway is activated, resulting in the phosphorylation of p27, a cyclin-dependent kinase inhibitor, at Thr 198. Phosphorylation of p27 increases its stability, and this permits the cell to survive growth factor withdrawal through autophagy. In addition to these two major regulatory pathways, other pathways and cell stress conditions have also been reported to participate in the regulation of autophagy, including the AKT/PKB pathway, the p52 pathway, the inositol pathway, endoplasmic reticulum stress, hypoxia, and the generation of reactive oxygen species (ROS) [30].

Stem cells are widely distributed in postnatal organs and tissues. In mammals, somatic stem cells play an essential role in development, tissue renewal, and certain disease processes. In contrast to the large amount of data derived from studies of somatic cells, cancer cells, and various disease models, the role of autophagy in the regulation of stem cell biology is poorly understood. It is accepted that the self-renewal and differentiation of stem cells require a strict control of protein turnover and lysosome-mediated degradation of the organelles [31]. Moreover, the autophagic process has been recently recognized as a major mechanism by which cells can attain their precise morphology and function, through the control of protein turnover [32]. Recent studies have shown that stem cell self-renewal and differentiation depend on the activation of autophagy [33, 34]. In response to the environment induction and the activation of hormones, autophagy can efficiently transport sets of transcription factors, adhesion molecules, or secreted factors, all of which are very important for stem cell self-renewal and differentiation.

**Figure 2:**
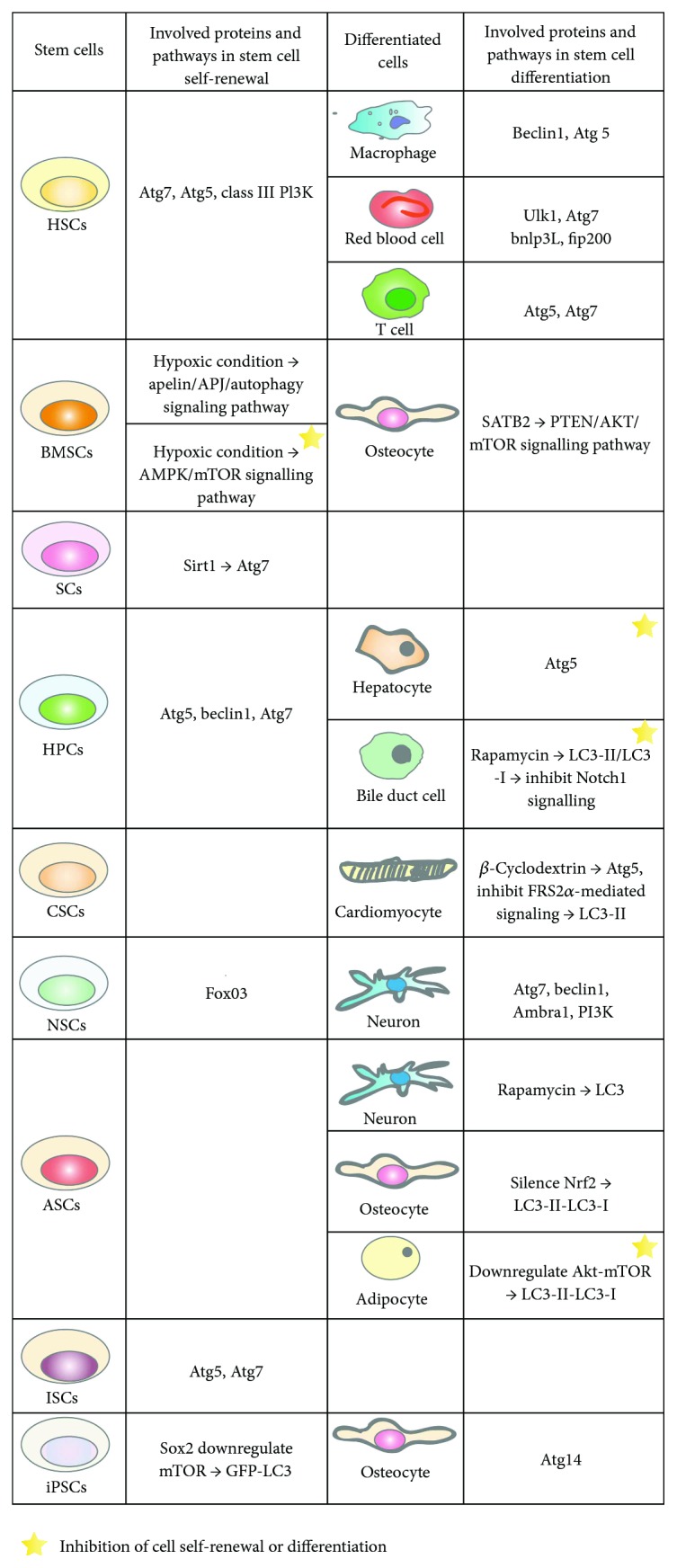
Autophagy involvement in stem cell's self-renewal and differentiation. HSCs: hematopoietic stem cells; BMSCs: bone marrow-derived mesenchymal stem cells; SCs: satellite cells; HPCs: hepatic progenitor cells; CSCs: cardiac stem cells; NSCs: neural stem cells; ASCs: adipose-derived stem cells; ISCs: intestinal stem cells; iPSCs: induced pluripotent stem cells.

Thus, autophagy is expected to play an important role in the regulation of stem cell biology. In this review, we discuss current knowledge from a range of different stem cell systems that significantly advance our understanding of the role of autophagy in stem cell biology (Figure 2).

## 2. Autophagy in Hematopoietic Stem Cells

Hematopoietic stem cells (HSCs) are the stem cells that give rise to all blood cells through the process of hematopoiesis. The continued maintenance of blood cells is ensured by a pool of HSCs that reside in hypoxic niches in the bone marrow [35, 36]. Recent works suggested that autophagic mechanisms are highly active in HSCs [37]. HSCs can quickly turn on the autophagic process to allow them to cope with cellular stresses, orchestrated by forkhead box O3 (FoxO3, a transcription factor) [38] or in response to increased metabolic load through the induction of parkin-dependent mitophagy [39].

Autophagy has been reported to be indispensable during the self-renewal of HSCs. One *in vitro* study revealed that human adult HSCs fail to form colonies in colony-forming assays when autophagy is inhibited using 3-methyladenine (3-MA), an autophagy inhibitor that targets phosphatidylinositol 3-kinase (PI3K), or an siRNA targeted to ATG5 [33]. In the hematopoietic system, loss of the essential autophagy gene Atg7 or Atg5 impairs HSC function, leading to severe myeloproliferation and bone marrow failure [38, 39]. Moreover, deleting the essential autophagy gene Atg7 in the hematopoietic system results in HSCs that have an accumulation of mitochondria and ROS, as well as increased proliferation and DNA damage [38]. These findings indicated the essential role of autophagy in the maintenance of HSCs.

Autophagy has also been reported to positively regulate HSC differentiation. Autophagy prevents apoptosis during the cell differentiation process, by preventing ROS generation, ER stress, and DNA damage. For example, monocytes, which are derived from HSCs, eventually differentiate into macrophages or dendritic cells [40]. However, monocytes are programmed to undergo apoptosis in the absence of stimulation [41], and the monocyte-macrophage differentiation stimuli not only cause cellular changes but also prevent the default apoptosis of monocytes [42]. Zhang et al. have demonstrated that autophagy is induced when monocytes are triggered to differentiate. A differentiation signal releases beclin1 from Bcl-2 by activating JNK and blocks Atg5 cleavage, thereby inducing autophagy. Furthermore, this induction of autophagy is critical for the survival and differentiation of monocytes. Inhibition of autophagy also results in the apoptosis of cells that are undergoing differentiation [42]. This finding indicates that induction of autophagy is essential for monocyte-macrophage differentiation. Clearance of organelles is also an important process in the regulation of HSC differentiation. During red blood cell differentiation, the nucleus is expelled from the cell, whereas mitochondria are cleared by means of mitophagy [43]. Targeted deletion of autophagy genes, including Ulk1 [44], Atg7 [45], Bnip3L [46], and Fip200 [47], caused defective erythroid differentiation and anemia. Metabolic adaption is linked to autophagy by providing the nutrients and ATP necessary for differentiation. Xu et al. have shown that autophagy decreased in activated proliferating effector CD8+ T cells and was then upregulated when the cells stopped dividing. Deletion of the autophagy-related molecule Atg5 or Atg7 has little to no effect on the proliferation and function of these effector T cells, but these autophagy-deficient effector cells had survival defects that resulted in the compromised formation of memory T cells, indicating that autophagy is needed during the differentiation of memory T cells [48].

## 3. Autophagy in Bone Marrow-Derived Mesenchymal Stem Cells

Bone marrow-derived mesenchymal stem cells (BMSCs) are pluripotent adult stem cells that are capable of differentiating into diverse cell types, including osteocytes, adipocytes, endothelial cells, cardiomyocytes, and neurons, when exposed to the appropriate signals [49].

Studies have suggested that autophagy can induce BMSC apoptosis or promote BMSC proliferation. Recently, several studies have also shown that hypoxic conditions activate the BMSC autophagic flux through the AMPK/mTOR pathway, and this activation of autophagy contributes to hypoxia-induced apoptosis. In this study, the number of TUNEL-positive cells decreased in the presence of the autophagy inhibitor 3-MA, whereas the number of TUNEL-positive cells was increased by the autophagy inducer rapamycin. In this study, the authors measured autophagy induction by LC3 formation, which was shown to be blocked by 3-MA and increased by rapamycin under hypoxic conditions [50, 51]. An opposing study from Li et al. found that autophagy is involved in hypoxia-inducted BMSC proliferation. These authors showed that hypoxia induces the proliferation of BMSCs through the activation of the apelin/APJ/autophagy signaling pathway [52]. On the other hand, other studies have suggested that autophagy is important for preventing senescence in BMSCs. Compared to tibia-derived BMSCs (T-BMSCs), mandible-derived BMSCs (M-BMSCs) were reported to have higher levels of expression of the special AT-rich sequence-binding protein 2 (SATB2) and stemness markers (such as NANOG, OCT-4, SOX2, and NESTIN); however, they also exhibited higher degrees of autophagy and a greater resistance to aging under normal or hypoxic/serum deprivation conditions [53].

Autophagy also promotes BMSC differentiation into the osteoblastic lineage. Nuschke et al. have recently demonstrated that undifferentiated BMSCs accumulate nondegraded autophagic vacuoles, with little autophagic turnover, whereas stimulation of osteogenic differentiation leads to a consistent increase in autophagic turnover. In addition, SATB2, an AT-rich DNA-binding protein, has the ability to promote osteogenic differentiation and bone defect regeneration in BMSCs, and this is thought to occur through the upregulation of pluripotency genes and autophagy-related genes, which, in turn, activate the PTEN/AKT/mTOR signaling pathway [54].

## 4. Autophagy in Skeletal Muscle Stem Cells

Skeletal muscle stem cells or satellite cells (SCs) are located between the basement membrane and the sarcolemma in muscle fibers and are responsible for the growth and regeneration of muscle fibers following injury or disease [55, 56].

Autophagy has been found to play a positive role in maintaining the stemness status of SCs. Satellite cells are usually in the quiescent state, but they can be stimulated to enter the proliferative state when exposed to environmental stimuli [57]. In this context, autophagy was demonstrated to operate in two different scenarios. In the first scenario, a recent study has reported that autophagy was induced during SC activation. Specifically, this study has proposed that autophagy, induced by Sirt1 during SC activation, provides the nutrients necessary to meet the bioenergetic requirements for the transition of SCs from the quiescent state to the activated state during muscle injury. This study also proposed that a relative lack of nutrient availability induces autophagy by deacetylating ATG7 during the activation phase [58]. Of note, this study proposed that a relative lack of nutrient availability induces autophagy during the satellite cell activation phase, mimicking starvation-induced autophagy, a process necessary for cellular adaptation to nutritional stress. In the second scenario, autophagy maintains stemness by preventing senescence. García-Prat et al. have reported that young quiescent SCs have a basal autophagic flux in resting muscle and that this basal activity helps to preserve the integrity and fitness of the muscle fibers. These studies also revealed that the regenerative function of SCs declines during aging, owing mainly to the transition from a normal quiescent state into an irreversible senescent state [59, 60]. The physiological decline of autophagy in older SCs, or in genetically impaired young cells, can result in toxic cellular waste accumulation, which causes an entry into senescence and a decline in the function and number of SCs. However, a reestablishment of autophagy can reverse the senescent state and restore the regenerative function of geriatric SCs [60]. Thus, autophagy is required for the homeostatic maintenance of SCs under normal physiological conditions as well as during aging.

Active autophagy is coupled with the regeneration of dystrophic muscles. Stimulating autophagy enhances adult SC activation and proliferation, whereas inhibition of autophagy leads to a complete impairment of both processes. Interventions that extend the activation of autophagy might be beneficial in the treatment of Duchenne muscular dystrophy [61]. Thus, autophagy could be used as a “disease modifier” whereby a treatment that increases autophagy could promote muscle regeneration and delay disease progression.

## 5. Autophagy in Hepatic Progenitor Cells

The liver is unique in its extraordinary capacity to regenerate following a variety of injuries. Studies have shown that the regenerative ability of the liver can be mainly attributed to resident hepatic progenitor cells (HPCs), which are defined as cells that give rise to both hepatocytes and biliary epithelial cells (cholangiocytes) following liver injury [62]. While the role of autophagy in the regulation of hepatocytes has been wildly studied during liver regeneration [63] and the maintenance of liver metabolic homeostasis [64], there is very little knowledge available concerning the role of autophagy in HPCs.

It has been reported that inhibition of autophagy by knockdown of the essential autophagy gene Atg5 or beclin1 (Becn1) impaired the clonogenic and proliferative capability of HPCs. In this study, the efficiency of hepatic progenitor cell (HPC) self-renewal was assessed by the rate of colony formation using a colony-forming unit (CFU) assay. HPCs were infected with lentivirus expressing shNC or shRNA inhibiting Atg5 or Becn1. CFU numbers in shAtg5/shBecn1-HPCs were significantly decreased compared with those in shNC-HPCs. In addition, an *in vitro* proliferation assay demonstrated that the level of proliferation in shAtg5/shBecn1-HPCs was significantly lower than that in shNC-HPCs at 24, 48, and 72 hours after plating [65]. Similar results were found by Xue et al. who found that Atg7 or Atg5 inhibition reduced the colony- and spheroid-forming ability of HPCs. A deficiency in autophagy has also been shown to increase the accumulation of damaged mitochondria and mitochondrial reactive oxygen species (mtROS) and suppress the homologous recombination (HR) pathway for DNA damage repair in HPCs [66]. These results demonstrated that autophagy plays an indispensable role in stemness-associated expansion.

As far as current studies have reported, autophagy plays a negative role in the process of HPC differentiation. Zeng et al. have demonstrated that autophagy, detected by an increase in the LC3-II/LC3-I ratio, is decreased during the early stage of biliary differentiation of HPCs and is then maintained at a low level at later stages in the differentiation process [67]. To investigate whether induction of autophagy has an effect on the biliary differentiation of HPCs, they have examined the effect of two autophagy stimuli, the mTOR inhibitor rapamycin and starvation. Activation of autophagy by rapamycin or starvation suppressed the biliary differentiation of WB-F344 cells and led to the increase in the LC3-II/LC3-I ratio and in P62 levels [67]. They also have reported that autophagy inhibits the Notch1 signaling pathway, which contributed to biliary differentiation and morphogenesis. These results demonstrate that autophagy regulates biliary differentiation of hepatic progenitor cells through the Notch1 signaling pathway [67]. The effects of autophagy, p62, and related signaling pathways on hepatic differentiation were further investigated. Sugiyama et al. have reported that silencing the genes for ATG5 and/or SQSTM1/p62 promotes the amino acid activation of the mTOR pathway, indicating that promoting the amino acid sensitivity of the mTOR pathway is dependent on p62 accumulated by inhibition of autophagy and that this process plays an important role in the hepatic differentiation of stem/progenitor cells [68].

## 6. Autophagy in Cardiac Stem Cells

Characterized by the death of cardiomyocytes, heart failure remains one of the leading causes of death in the world [69]. Mobilizing heart endogenous cardiac stem cells (CSCs) to differentiate into myocardial cells is a new strategy that is being attempted to treat heart failure [70, 71].

Increased cardiac differentiation is associated with decreased proliferation of cardiomyocytes [72]. The role of autophagy in facilitating differentiation of CSCs was initially recognized by Zhang and his colleagues [73]. In their study, the FGF signaling axis was reported to inhibit the premature differentiation of CSCs by suppressing autophagy. The Wnt signaling pathway, an upstream regulator of the FGF pathway, also exerts an inhibitory effect on cardiac cell differentiation mediated through GSK3-TIP60-ULK1 signaling [73]. Shi et al. have revealed that changes in cholesterol metabolism (*β*-cyclodextrin) induce autophagy by increasing the expression of Atg5 and also trigger myocardial differentiation of CSCs. This process was characterized by the activation of the JNK/STAT3 and GSK3*β*/*β*-catenin pathways, followed by the increased expression of cardiac transcription factors (Nkx2.5 and GATA4), structural proteins (e.g., cardiac troponin T), and transcriptional enhancers (e.g., Mef2c) and an induction of GATA4 translocation to the cell nucleus [74]. Zhang et al. have investigated the mechanism by which FGF signaling regulates CSC differentiation and demonstrated that disruption of FGF signaling leads to the premature differentiation of CSCs in mice. Moreover, they also reported that inhibiting FRS2*α*-mediated signaling increases autophagy by increasing LC3-II levels and promotes the myocardial differentiation of CSCs and vice versa, indicating the positive role of autophagy in CSC differentiation [72].

## 7. Autophagy in Neural Stem Cells

As discussed above, autophagy is a metabolic mechanism that maintains cellular homeostasis, through which the metabolic needs of cells and the renewal of organelles can be met. Because a defect in autophagy results in altered protein turnover, or the accumulation of misfolded proteins, this could underlie a number of neurodegenerative diseases, such as Alzheimer's disease, Parkinson's disease, and Huntington's disease [75, 76]. Neural stem cells (NSCs) are self-renewing, multipotent cells that are present in neurogenic niches in the brain and are responsible for generating the neuronal and glial cells in the nervous system. Recent studies have shown that autophagy is involved in the regulation of stemness and neurogenesis in neural stem cells (NSCs) [77, 78].

Autophagy defects may lead to defective self-renewal of NSCs. For example, activation of the FOXO family (e.g., FOXO1 and FOXO3) of transcription factors has been reported to be involved in the activation of autophagy in cancer cells, as well as in muscle [79, 80]. In addition, inactivation of FOXO1, FOXO3, and FOXO4 (or FOXO3 only [81]) results in defective self-renewal and differentiation of NSCs, paralleled by increased ROS production [82]. These findings raise the interesting possibility that autophagy defects in these mice might contribute to the ROS elevation caused by FOXO deficiency, thus leading to defective self-renewal of NSCs.

There is also evidence for an active role for autophagy during NSC differentiation. During differentiation, NSCs need to remodel their cytoskeleton and shape in an energy-consuming process. The capacity of autophagy to recycle cellular components and provide energy could fulfill these requirements, thus supporting differentiation. Vázquez et al. have reported an increase in the expression of the autophagy genes Atg7, Becn1, Ambra1, and LC3 in the mouse embryonic olfactory bulb during the initial period of neuronal differentiation, along with a parallel increase in neuronal markers, while pharmacological inhibition of autophagy with 3-MA or wortmannin markedly decreased neurogenesis in mice, supporting the role of autophagy in neuronal differentiation. This study indicates a homeostatic role for autophagy as an energy provider during the early stages of neuronal differentiation [83]. Fimia et al. have also shown that the Ambra1 (activating molecule in beclin1-regulated autophagy) knockout in mouse embryos leads to severe neural tube defects associated with autophagy impairment, the accumulation of ubiquitinated proteins, unbalanced cell proliferation, and excessive cell death [84].

Taken together, all these observations point to the concept that autophagy plays a supporting role in the proliferation and neuronal differentiation of NSCs.

## 8. Autophagy in Adipose-Derived Stem Cells

Being easily harvested from adipose tissue and abundant in number, adipose-derived stem cells (ASCs) are among the most promising sources of MSCs available.

Recent studies have shown that autophagy can work either as a promoter or as a suppressor in the ASC differentiation process. Lu and his colleagues have demonstrated the positive role of autophagy in the process of ASC differentiation into neuronal-like cells. Their data revealed that the ASCs exhibit a neuronal-like morphology and a significantly increased differentiation rate after rapamycin induction (200 *μ*g/L) compared to the control. Moreover, expression of the autophagy protein (LC3) was also significantly upregulated with respect to untreated cells [85]. Zhao et al. have investigated the negative role of autophagy in the adipogenic differentiation of hASCs and demonstrated that gamma-tocotrienol specifically inhibited the early stages of adipogenic differentiation of hASCs. Importantly, this process is regulated by activation of autophagy, as shown by increases in autophagic flux and cytosolic autophagosome (LC3II) accumulation [86]. Bo et al. also discovered that autophagy plays a negative regulatory role in adipogenic differentiation. Fluoxetine, a drug used to treat obesity, has been shown to inhibit the proliferation and adipogenic differentiation of ASCs, likely through increasing the expression of the autophagy-related genes, SQSTM1 and LC3II [87]. Ejaz et al. identified DIRAS3 and IGF-1 as target genes that were upregulated in ASCs derived from the subcutaneous white adipose tissue of long-term weight loss patients. Moreover, DIRAS3 downregulates Akt-mTOR signaling in ASCs and inhibits adipogenesis and activates autophagy in these cells [88].

The relationship between nuclear factor erythroid 2-related factor 2 (Nrf2) and autophagy has been investigated extensively. Nrf2 is a transcriptional factor that promotes cell survival and protects cells against oxidative stress-induced damage [89]. Nrf2 is negatively regulated by Kelch-like ECH-associated protein 1 (Keap-1), which binds to Nrf2 in the cytoplasm and directs it for proteasomal degradation [90]. The p62/sequestosome 1 (SQSTM1) protein acts as a cargo receptor for autophagic degradation of ubiquitinated targets. Induction of the p62 gene by oxidative stress is mediated by Nrf2, and, at the same time, the p62 protein contributes to the activation of Nrf2. In addition, p62 docks and binds directly to Keap-1 via a motif designated in the Keap-1 interacting region (KIR). The binding of p62 to Keap-1 blocks the interaction between Keap-1 and Nrf2, and then Nrf2 goes to the nucleus and facilitates the activation of Nrf2 target genes [91–93]. Thus, p62 contributes to the activation of Nrf2 target genes in response to oxidative stress by creating a positive feedback loop. Tao et al. have explored the involvement of the Nrf2 pathway and autophagy on the osteogenic differentiation of ASCs under oxidative stress conditions [94]. They found that exposure of ASCs to H_2_O_2_ led to the induction of apoptosis and autophagy, the upregulation of Nrf2, and the promotion of osteogenesis. In contrast, suppression of autophagic activity resulted in the activation of the Nrf2 pathway and the inhibition of osteoblastic differentiation of ASCs upon ROS stimulation. Silencing of Nrf2 has been shown to promote autophagy and the osteoblastic differentiation of ASCs upon ROS stimulation [94]. These findings indicate that oxidative stress induces autophagy and promotes osteoblastic differentiation of ADSCs, and these effects are enhanced by the silencing of Nrf2, suggesting that a negative interaction between the Nrf2 pathway and autophagy may modulate oxidative stress-induced ASC osteogenesis.

## 9. Autophagy in Intestinal Stem Cells

Throughout life, the intestinal tract undergoes a continual and rapid turnover of epithelial cells. Studies in both mice and humans have shown that this process is regulated and maintained by a population of intestinal stem cells (ISCs), which are capable of replenishing themselves and giving rise to all of the intestinal epithelial cell lineages [95].

Recent work has suggested that intrinsic autophagy is important for the maintenance of intestinal stem cells by reducing excessive reactive oxygen species. This stem cell maintenance is necessary to provide for damage-induced intestinal regeneration. Asano et al. have shown that intrinsic autophagy in ISCs is important for ISC maintenance by reducing excessive ROS. Mice lacking ATG5 in intestinal epithelial cells (iECs) had significantly fewer ISCs than did control mice and showed impaired ISC-dependent intestinal recovery after irradiation. Crypt ISCs from Atg5ΔIEC mice had significantly higher reactive oxygen species (ROS) levels than did those from control mice. A ROS-inducing reagent decreased the ISC number and impaired ISC regenerative capacity *in vitro*, and treating Atg5ΔIEC mice with an antioxidant rescued these defects [96]. Similar results were found by Shaffiey et al. They found that acute exposure to lipopolysaccharide (LPS) caused a significant reduction in the mRNA expression of cycling stem cell markers in both WT and ATG7ΔIEC mice; however, the changes were much more dramatic in ATG7ΔIEC mice. These phenomena suggested that autophagy may help intestinal repair through the regulation of ISCs [97].

Given that autophagy is essential for the recovery of iECs after irradiation or LPS treatment, optimizing autophagy, particularly in ISCs, might promote the recovery of iECs after injury and perhaps lead to an autophagy-based therapy.

## 10. Autophagy in Induced Pluripotent Stem Cells

Induced pluripotent stem cells (also known as iPS cells or iPSCs) are a type of pluripotent stem cells that can be generated from adult (nonpluripotent) cells. They not only bypass the need for embryos but can be made in a patient-matched manner, holding a great promise in the field of regenerative medicine [98]. iPSCs are typically obtained by introducing a specific set of pluripotency-associated genes into adult cells. The original set of reprogramming factors (also dubbed Yamanaka factors) used for the productions of iPSCs is the transcription factors Oct4 (Pou5f1), Sox2, cMyc, and Klf4. Upon introduction of these reprogramming factors, cells begin to form colonies that resemble pluripotent stem cells and can be isolated based on their morphology or surface markers.

Recent studies have shown that high levels of basal autophagy activity are present during iPSC derivation and maintenance. Successful generation of iPSCs entails a major metabolic switch from mitochondrial oxidative phosphorylation to glycolysis during the reprogramming process; this process is related to the mTOR signaling pathway. In particular, fine-tuning of mTOR signaling can affect mitochondrial dynamics to allow for the segregation of mitochondria that are destined for clearance through autophagy [99].

A further study has revealed that mTOR is downregulated by Sox2 at an early stage of iPSC generation and that this transient downregulation of mTOR is required for reprogramming to occur. In the absence of Sox2, mTOR remains at a high level and inhibits autophagy. This finding indicates that Sox2-dependent temporal regulation of autophagy is a key step in cellular reprogramming processes [100]. Canonical autophagy is mediated by the evolutionarily conserved autophagy-related genes, that is, Atg genes [101]. Atg5 has been characterized as being an essential component in canonical autophagy, such that Atg5 deletion completely inhibits autophagy [102, 103]. More recently, it was reported that iPSC reprogramming relies on the Atg5-dependent autophagy that is transiently activated by Sox2 overexpression early in reprogramming and that cells lacking Atg5 may abrogate iPSC formation [100]. However, discrepancy has been found with these findings. Sotthibundhu et al. have reported that robust iPSC reprogramming does not rely on Atg5-dependent canonical autophagy. This Atg5-independent autophagic process clears mitochondria to facilitate the metabolic switch from mitochondrial oxidative phosphorylation to glycolysis that has to occur during reprogramming. Blocking such autophagy, but not canonical autophagy, inhibits mitochondrial clearance, in turn, preventing iPSC induction. These results suggest that the Atg5-independent autophagy is crucial for establishing pluripotency [104].

Ozeki and his colleagues have recently investigated miR-211 regulation and Atg signaling during the osteogenic differentiation of human iPSCs [105]. During osteogenic differentiation, there were dramatic increases in the miR-211 and protein levels of Atg14, together with increases in the amount of autophagosomes and increases in autophagic fluxes in human iPSCs. Treatment with a small interfering RNA capable of targeting Atg14 suppressed the osteogenic differentiation of these human iPSCs. Importantly, the osteogenic phenotype was inhibited by chloroquine (an autophagy inhibitor) but was increased after treatment with rapamycin (an autophagy inducer). The addition of chloroquine resulted in the suppression of Atg14 expression and a decrease in autophagosomes in differentiated cells; in contrast, addition of rapamycin resulted in an increase in Atg14 expression and the accumulation of autophagosomes [105].

Neurodegenerative diseases originate from a loss of neurons in the central nervous system and are severely debilitating. Until recently, the main resource for *in vitro* neuronal studies has been primary neurons isolated from rodent brains. However, research focused on human neurons is restricted because primary human cultures are limited by sample availability and by obvious ethical concerns. The ability to differentiate hiPSCs into neurons has provided researchers with the tools to begin to study human neurodegenerative diseases. In the mammalian nervous system, autophagy is required to maintain its normal functions and homeostasis. Using hiPSC technology, researchers have been able to generate many types of neurons that are lost in human neurodegenerative disease in order to study the role of autophagy in these diseases [106].

Alzheimer's disease (AD) is the most common neurodegenerative disease [107]. The study of autophagy in iPSC-derived human AD neurons has improved our understanding of autophagy in this disease. Lee et al. have examined autophagy dysfunction in iPSC-derived neurons derived from familial AD (FAD) patient cells with a presenilin-1 (PS-1) mutation. They found an increase in autophagic vacuole accumulation in PS-1 mutant neurons and a decrease in TFEB target genes, indicative of decreased autophagic flux. In addition, when they suppressed acid sphingomyelinase, both lysosomal biogenesis and autophagy activity were restored to normal levels [108]. Reddy et al. have generated iPSC-derived human forebrain cortical neurons from AD patients with M146L and A246E mutations, as well as with a PS-1 knockdown in control neurons [109]. They found a reduction in the CLEAR-luciferase reporter activity in these iPSC-derived human AD neurons as well as a decrease in LC3II levels in PS-1-knockdown neurons, suggesting decreased autophagy initiation, as well as autophagic flux.

Parkinson's disease (PD) is a neurodegenerative disease, second only to AD, which is caused by the loss of dopaminergic (DA) neurons in the substantia nigra, leading to the disruption of the nigrostriatal pathway [110]. Autophagy flux has been studied in iPSC-derived DA neurons from patients with idiopathic PD (ID-PD) or familial PD (mutation in leucine-rich repeat kinase 2 (LRRK2)). Over long-time culture, dopaminergic neurons (DAn) differentiated from either ID-PD- or LRRK2-PD-iPSCs showed morphological alterations, including reduced numbers of neurites and neurite arborization, as well as accumulation of autophagic vacuoles, which were not evident in DAn differentiated from Ctrl-iPSC. Further induction of autophagy and/or inhibition of lysosomal proteolysis greatly exacerbated the DAn morphological alterations, indicating autophagic compromise in DAn from ID-PD- and LRRK2-PD-iPSCs [111]. Fernandes et al. generated midbrain DA neurons using iPSCs from PD patients with the GBA-N370S mutation [112]. They recorded increased autophagosome numbers associated with elevated beclin1 and P62/SQSTM1 levels in these GBA-N370S lines. These observations strongly suggested that autophagosomal-lysosomal turnover is impaired in the mutant lines.

Progress in the application of iPSC neural differentiation protocols has provided researchers with an unrivalled opportunity to study, in greater detail, how autophagy pathways contribute to neuronal function and survival in complex human neurodegenerative diseases and how these can be exploited for neuroprotective and/or neurorestorative therapies.

## 11. Concluding Remarks

Stem cells fuel tissue development, renewal, and regeneration, and these activities require a strict control of protein turnover and lysosomal digestion of organelles in stem cells. Autophagy is a highly conserved process and serves as a major regulator for the acquisition of precise cell morphology and function through the control of protein turnover. The past decade has witnessed a significant growth in interest regarding stem cells and autophagy; however, our understanding of the role of autophagy in stem cell biology is still in its infancy. Thus, it is reasonable to expect that a deeper understanding of the role of autophagy in stem cell biology may promote the research and application of stem cells on a broader scale. Given the different specific characteristics of particular stem cells, studies on the regulation of autophagy in stem cell biology will be facilitated by using well-defined *in vitro* stem cell systems and by using genetic models *in vivo*. In addition, it will still be necessary to develop specific methods to allow for the monitoring of selective autophagy targets (e.g., the mitochondrion) in living stem cells that are undergoing proliferation or differentiation, which will also help to increase our understanding of basic stem cell biology.

## References

[1] Klionsky D. J. (2004). Cell biology: regulated self-cannibalism. *Nature*.

[2] Finn P. F., Dice J. F. (2006). Proteolytic and lipolytic responses to starvation. *Nutrition*.

[3] Mizushima N., Levine B. (2010). Autophagy in mammalian development and differentiation. *Nature Cell Biology*.

[4] Mizushima N., Komatsu M. (2011). Autophagy: renovation of cells and tissues. *Cell*.

[5] Martinez-Lopez N., Athonvarangkul D., Singh R., Atzmon G. (2015). Autophagy and aging. *Longevity Genes. Advances in Experimental Medicine and Biology, vol 847*.

[6] Dong H., Czaja M. J. (2011). Regulation of lipid droplets by autophagy. *Trends in Endocrinology & Metabolism*.

[7] Mariño G., Niso-Santano M., Baehrecke E. H., Kroemer G. (2014). Self-consumption: the interplay of autophagy and apoptosis. *Nature Reviews Molecular Cell Biology*.

[8] Wen X., Klionsky D. J. (2016). Autophagy is a key factor in maintaining the regenerative capacity of muscle stem cells by promoting quiescence and preventing senescence. *Autophagy*.

[9] Gomes L. C., Scorrano L. (2013). Mitochondrial morphology in mitophagy and macroautophagy. *Biochimica et Biophysica Acta (BBA) - Molecular Cell Research*.

[10] Dubouloz F., Deloche O., Wanke V., Cameroni E., De V. C. (2005). The TOR and EGO protein complexes orchestrate microautophagy in yeast. *Molecular Cell*.

[11] Majeski A. E., Fred Dice J. (2004). Mechanisms of chaperone-mediated autophagy. *The International Journal of Biochemistry & Cell Biology*.

[12] Deretic V. (2005). Autophagy in innate and adaptive immunity. *Trends in Immunology*.

[13] Al Rawi S., Louvet-Vallée S., Djeddi A. (2011). Postfertilization autophagy of sperm organelles prevents paternal mitochondrial DNA transmission. *Science*.

[14] Farré J. C., Krick R., Subramani S., Thumm M. (2009). Turnover of organelles by autophagy in yeast. *Current Opinion in Cell Biology*.

[15] Monastyrska I., Klionsky D. J. (2006). Autophagy in organelle homeostasis: peroxisome turnover. *Molecular Aspects of Medicine*.

[16] Ganle I. G., Lam D. H., Wang J. (2009). ULK1·ATG13·FIP200 complex mediates mTOR signaling and is essential for autophagy. *The Journal of Biological Chemistry*.

[17] Chan E. Y. (2009). mTORC1 phosphorylates the ULK1-mAtg13-FIP200 autophagy regulatory complex. *Science Signaling*.

[18] Kim J., Kim Y. C., Fang C. (2013). Differential regulation of distinct Vps34 complexes by AMPK in nutrient stress and autophagy. *Cell*.

[19] Yuan H. X., Russell R. C., Guan K. L. (2013). Regulation of PIK3C3/VPS34 complexes by MTOR in nutrient stress-induced autophagy. *Autophagy*.

[20] Kraft C., Martens S. (2012). Mechanisms and regulation of autophagosome formation. *Current Opinion in Cell Biology*.

[21] Kabeya Y., Mizushima N. A., Oshitani O. S., Ohsumi Y., Yoshimori T. (2004). LC3, GABARAP and GATE16 localize to autophagosomal membrane depending on form-II formation. *Journal of Cell Science*.

[22] Kabeya Y., Mizushima N., Ueno T. (2000). LC3, a mammalian homologue of yeast Apg8p, is localized in autophagosome membranes after processing. *The EMBO Journal*.

[23] Mizushima N., Noda T., Yoshimori T. (1998). A protein conjugation system essential for autophagy. *Nature*.

[24] Mizushima N., Noda T., Ohsumi Y. (1999). Apg16p is required for the function of the Apg12p-Apg5p conjugate in the yeast autophagy pathway. *The EMBO Journal*.

[25] Yoshimori T. (2004). Autophagy: a regulated bulk degradation process inside cells. *Biochemical and Biophysical Research Communications*.

[26] Ichimura Y., Kirisako T., Takao T. (2000). A ubiquitin-like system mediates protein lipidation. *Nature*.

[27] Srinivas V., Bohensky J., Shapiro I. M. (2008). Autophagy: a new phase in the maturation of growth plate chondrocytes is regulated by HIF, mTOR and AMP kinase. *Cells, Tissues, Organs*.

[28] Castets P., Rüegg M. A. (2013). MTORC1 determines autophagy through ULK1 regulation in skeletal muscle. *Autophagy*.

[29] Song L., Su M., Wang S. (2014). MiR-451 is decreased in hypertrophic cardiomyopathy and regulates autophagy by targeting TSC1. *Journal of Cellular and Molecular Medicine*.

[30] Tekirdag K. A., Ozturk D. G., Gozuacik D. (2015). Chapter 4 – regulation of autophagy by microRNAs. *Autophagy: Cancer Other Pathologies Inflammation Immunity Infection and Aging*.

[31] Zeng M., Zhou J. N. (2008). Roles of autophagy and mTOR signaling in neuronal differentiation of mouse neuroblastoma cells. *Cellular Signalling*.

[32] Lum J. J., Deberardinis R. J., Thompson C. B. (2005). Autophagy in metazoans: cell survival in the land of plenty. *Nature Reviews Molecular Cell Biology*.

[33] Kanchan P., Scarth W. A., Katharina S. A. (2013). Tightrope act: autophagy in stem cell renewal, differentiation, proliferation, and aging. *Cellular and Molecular Life Sciences*.

[34] Vessoni A. T., Muotri A. R., Okamoto O. K. (2012). Autophagy in stem cell maintenance and differentiation. *Stem Cells and Development*.

[35] Suda T., Takubo K., Semenza G. L. (2011). Metabolic regulation of hematopoietic stem cells in the hypoxic niche. *Cell Stem Cell*.

[36] Gomez-Puerto M. C., Folkerts H., Wierenga A. T. J. (2016). Autophagy proteins ATG5 and ATG7 are essential for the maintenance of human CD34^+^ hematopoietic stem-progenitor cells. *Stem Cells*.

[37] Nguyen-McCarty M., Klein P. S. (2017). Autophagy is a signature of a signaling network that maintains hematopoietic stem cells. *PloS One*.

[38] Warr M. R., Binnewies M., Flach J. (2013). FOXO3A directs a protective autophagy program in haematopoietic stem cells. *Nature*.

[39] Ito K., Turcotte R., Cui J. (2016). Self-renewal of a purified *Tie2*^+^ hematopoietic stem cell population relies on mitochondrial clearance. *Science*.

[40] Dutta P., Nahrendorf M. (2014). Regulation and consequences of monocytosis. *Immunological Reviews*.

[41] Um H. D., Orenstein J. M., Wahl S. M. (1996). Fas mediates apoptosis in human monocytes by a reactive oxygen intermediate dependent pathway. *The Journal of Immunology*.

[42] Zhang Y., Morgan M. J., Chen K., Choksi S., Liu Z. G. (2012). Induction of autophagy is essential for monocyte-macrophage differentiation. *Blood*.

[43] Zhang J., Wu K., Xiao X. (2015). Autophagy as a regulatory component of erythropoiesis. *International Journal of Molecular Sciences*.

[44] Kundu M., Lindsten T., Yang C. Y. (2008). Ulk1 plays a critical role in the autophagic clearance of mitochondria and ribosomes during reticulocyte maturation. *Blood*.

[45] Mortensen M., Soilleux E. J., Djordjevic G. (2011). The autophagy protein Atg7 is essential for hematopoietic stem cell maintenance. *Journal of Experimental Medicine*.

[46] Sandoval H., Thiagarajan P., Dasgupta S. K. (2008). Essential role for Nix in autophagic maturation of erythroid cells. *Nature*.

[47] Liu F., Lee J. Y., Wei H. (2010). FIP200 is required for the cell-autonomous maintenance of fetal hematopoietic stem cells. *Blood*.

[48] Xu X., Araki K., Li S. (2014). Autophagy is essential for effector CD8 T cell survival and memory formation. *Nature Immunology*.

[49] Zhang F., Wang C., Jing S. (2013). Lectin-like oxidized LDL receptor-1 expresses in mouse bone marrow-derived mesenchymal stem cells and stimulates their proliferation. *Experimental Cell Research*.

[50] Molaei S., Roudkenar M. H., Amiri F. (2015). Down-regulation of the autophagy gene, *ATG7*, protects bone marrow-derived mesenchymal stem cells from stressful conditions. *Blood Research*.

[51] Zhang Z., Yang M., Wang Y. (2016). Autophagy regulates the apoptosis of bone marrow-derived mesenchymal stem cells under hypoxic condition via AMP-activated protein kinase/mammalian target of rapamycin pathway. *Cell Biology International*.

[52] Li L., Li L., Zhang Z., Jiang Z. (2015). Hypoxia promotes bone marrow-derived mesenchymal stem cell proliferation through apelin/APJ/autophagy pathway. *Acta Biochimica et Biophysica Sinica*.

[53] Dong W., Zhang P., Fu Y. (2015). Roles of SATB2 in site-specific stemness, autophagy and senescence of bone marrow mesenchymal stem cells. *Journal of Cellular Physiology*.

[54] Nuschke A., Rodrigues M., Stolz D. B., Chu C. T., Griffith L., Wells A. (2014). Human mesenchymal stem cells/multipotent stromal cells consume accumulated autophagosomes early in differentiation. *Stem Cell Research & Therapy*.

[55] Brack A. S., Rando T. A. (2012). Tissue-specific stem cells: lessons from the skeletal muscle satellite cell. *Cell Stem Cell*.

[56] Yin H., Price F., Rudnicki M. A. (2013). Satellite cells and the muscle stem cell niche. *Physiological Reviews*.

[57] Cheung T. H., Rando T. A. (2013). Molecular regulation of stem cell quiescence. *Nature Reviews: Molecular Cell Biology*.

[58] Tang A. H., Rando T. A. (2014). Induction of autophagy supports the bioenergetic demands of quiescent muscle stem cell activation. *The EMBO Journal*.

[59] García-Prat L., Martínez-Vicente M., Perdiguero E. (2016). Autophagy maintains stemness by preventing senescence. *Nature*.

[60] García-Prat L., Muñozcánoves P., Martínezvicente M., Perdiguero E., Cornelison D. (2017). Monitoring autophagy in muscle stem cells. *Muscle Stem Cells. Methods in Molecular Biology, vol 1556*.

[61] Fiacco E., Castagnetti F., Bianconi V. (2016). Autophagy regulates satellite cell ability to regenerate normal and dystrophic muscles. *Cell Death and Differentiation*.

[62] Miyajima A., Tanaka M., Itoh T. (2014). Stem/progenitor cells in liver development, homeostasis, regeneration, and reprogramming. *Cell Stem Cell*.

[63] Toshima T., Shirabe K., Fukuhara T. (2014). Suppression of autophagy during liver regeneration impairs energy charge and hepatocyte senescence in mice. *Hepatology*.

[64] Uchiyama Y., Kominami E. (2013). Autophagy regulates lipid droplet formation and adipogenesis. *Lipid Metabolism*.

[65] Cheng Y., Wang B., Zhou H. (2015). Autophagy is required for the maintenance of liver progenitor cell functionality. *Cellular Physiology & Biochemistry*.

[66] Xue F., Hu L., Ge R. (2016). Autophagy-deficiency in hepatic progenitor cells leads to the defects of stemness and enhances susceptibility to neoplastic transformation. *Cancer Letters*.

[67] Zeng J., Jing Y., Shi R. (2016). Autophagy regulates biliary differentiation of hepatic progenitor cells through Notch1 signaling pathway. *Cell Cycle*.

[68] Sugiyama M., Yoshizumi T., Yoshida Y. (2017). p62 promotes amino acid sensitivity of mTOR pathway and hepatic differentiation in adult liver stem/progenitor cells. *Journal of Cellular Physiology*.

[69] Braunwald E., Bristow M. R. (2000). Congestive heart failure: fifty years of progress. *Circulation*.

[70] Mayfield A. E., Tilokee E. L., Davis D. R. (2014). Resident cardiac stem cells and their role in stem cell therapies for myocardial repair. *Canadian Journal of Cardiology*.

[71] Fanton Y., Robic B., Rummens J. L. (2015). Cardiac atrial appendage stem cells engraft and differentiate into cardiomyocytes *in vivo*: a new tool for cardiac repair after MI. *International Journal of Cardiology*.

[72] Zhang J., Liu J., Huang Y. (2012). FRS2α-mediated FGF signals suppress premature differentiation of cardiac stem cells through regulating autophagy activity. *Circulation Research*.

[73] Zhang J., Liu J., Liu L., Mckeehan W. L., Wang F. (2012). The fibroblast growth factor signaling axis controls cardiac stem cell differentiation through regulating autophagy. *Autophagy*.

[74] Shi X., Li W., Liu H., Yin D., Zhao J. (2017). β-Cyclodextrin induces the differentiation of resident cardiac stem cells to cardiomyocytes through autophagy. *Biochimica et Biophysica Acta (BBA) - Molecular Cell Research*.

[75] Navone F., Genevini P., Borgese N. (2015). Autophagy and neurodegeneration: insights from a cultured cell model of ALS. *Cells*.

[76] Yoshimitsu K., Hiromi N. (2015). The function of autophagy in neurodegenerative diseases. *International Journal of Molecular Sciences*.

[77] Kempermann G., Gage F. H. (1999). New nerve cells for the adult brain. *Scientific American*.

[78] Doetsch F. (2003). A niche for adult neural stem cells. *Current Opinion in Genetics and Development*.

[79] Zhao Y., Yang J., Liao W. (2010). Cytosolic FoxO1 is essential for the induction of autophagy and tumour suppressor activity. *Nature Cell Biology*.

[80] Zhao J., Brault J. J., Schild A. (2007). FoxO3 coordinately activates protein degradation by the autophagic/lysosomal and proteasomal pathways in atrophying muscle cells. *Cell Metabolism*.

[81] Renault V. M., Rafalski V. A., Morgan A. A. (2009). FoxO3 regulates neural stem cell homeostasis. *Cell Stem Cell*.

[82] Paik J., Ding Z., Narurkar R. (2009). FoxOs cooperatively regulate diverse pathways governing neural stem cell homeostasis. *Cell Stem Cell*.

[83] Vázquez P., Arroba A. I., Cecconi F., de la Rosa E. J., Boya P., de Pablo F. (2012). Atg5 and Ambra1 differentially modulate neurogenesis in neural stem cells. *Autophagy*.

[84] Fimia G. M., Stoykova A., Romagnoli A. (2007). Ambra1 regulates autophagy and development of the nervous system. *Nature*.

[85] Lu Y., Yuan X., Sun Q., Ou Y. (2013). Autophagy activator promotes neuronal differentiation of adult adipose-derived stromal cells. *Neural Regeneration Research*.

[86] Zhao L., Ha J. H., Okla M., Chung S. (2014). Activation of autophagy and AMPK by gamma-tocotrienol suppresses the adipogenesis in human adipose derived stem cells. *Molecular Nutrition & Food Research*.

[87] Bo K. S., Ji H. K., Choi J. S., Hwang S. J., Sung J. H. (2015). Fluoxetine decreases the proliferation and adipogenic differentiation of human adipose-derived stem cells. *International Journal of Molecular Sciences*.

[88] Ejaz A., Mitterberger M. C., Lu Z. (2016). Weight loss upregulates the small GTPase DIRAS3 in human white adipose progenitor cells, which negatively regulates adipogenesis and activates autophagy via Akt–mTOR inhibition. *Ebiomedicine*.

[89] Ma Q. (2013). Role of Nrf2 in oxidative stress and toxicity. *Annual Review of Pharmacology and Toxicology*.

[90] Itoh K., Wakabayashi N., Katoh Y. (1999). Keap1 represses nuclear activation of antioxidant responsive elements by Nrf2 through binding to the amino-terminal Neh2 domain. *Genes & Development*.

[91] Simon H. U., Friis R., Tait S. W. G., Ryan K. M. (2017). Retrograde signaling from autophagy modulates stress responses. *Science Signaling*.

[92] Jain A., Lamark T., Sjøttem E. (2010). *p62/SQSTM1* is a target gene for transcription factor NRF2 and creates a positive feedback loop by inducing antioxidant response element-driven gene transcription. *The Journal of Biological Chemistry*.

[93] Komatsu M., Kurokawa H., Waguri S. (2010). The selective autophagy substrate p62 activates the stress responsive transcription factor Nrf2 through inactivation of Keap1. *Nature Cell Biology*.

[94] Tao J., Wang H., Zhai Y. (2016). Downregulation of Nrf2 promotes autophagy-dependent osteoblastic differentiation of adipose-derived mesenchymal stem cells. *Experimental Cell Research*.

[95] Scoville D. H., Sato T., He X. C., Li L. (2008). Current view: intestinal stem cells and signaling. *Gastroenterology*.

[96] Asano J., Sato T., Ichinose S. (2017). Intrinsic autophagy is required for the maintenance of intestinal stem cells and for irradiation-induced intestinal regeneration. *Cell Reports*.

[97] Shaffiey S., Sodhi C., Jia H. (2014). A novel role of autophagy in intestinal epithelial stem cell proliferation and renewal. *Journal of Surgical Research*.

[98] Takahashi K., Tanabe K., Ohnuki M. (2010). Induction of pluripotent stem cells from adult human fibroblasts by defined factors. *Nederlands Tijdschrift voor Geneeskunde*.

[99] Menendez J. A., Vellon L., Oliveras-Ferraros C., Cufí S., Vazquez-Martin A. (2011). mTOR-regulated senescence and autophagy during reprogramming of somatic cells to pluripotency: a roadmap from energy metabolism to stem cell renewal and aging. *Cell Cycle*.

[100] Wang S., Xia P., Ye B., Huang G., Liu J., Fan Z. (2013). Transient activation of autophagy via Sox2-mediated suppression of mTOR is an important early step in reprogramming to pluripotency. *Cell Stem Cell*.

[101] Boya P., Reggiori F., Codogno P. (2013). Emerging regulation and functions of autophagy. *Nature Cell Biology*.

[102] Mizushima N., Yamamoto A., Hatano M. (2001). Dissection of autophagosome formation using Apg5-deficient mouse embryonic stem cells. *The Journal of Cell Biology*.

[103] Kuma A., Hatano M., Matsui M. (2004). The role of autophagy during the early neonatal starvation period. *Nature*.

[104] Sotthibundhu A., McDonagh K., von Kriegsheim A. (2016). Rapamycin regulates autophagy and cell adhesion in induced pluripotent stem cells. *Stem Cell Research & Therapy*.

[105] Ozeki N., Hase N., Hiyama T. (2017). MicroRNA-211 and autophagy-related gene 14 signaling regulate osteoblast-like cell differentiation of human induced pluripotent stem cells. *Experimental Cell Research*.

[106] Menzies F. M., Fleming A., Caricasole A. (2017). Autophagy and neurodegeneration: pathogenic mechanisms and therapeutic opportunities. *Neuron*.

[107] Mohandas E., Rajmohan V., Raghunath B. (2009). Neurobiology of Alzheimer’s disease. *Indian Journal of Psychiatry*.

[108] Lee J. K., Jin H. K., Park M. H. (2014). Acid sphingomyelinase modulates the autophagic process by controlling lysosomal biogenesis in Alzheimer’s disease. *Journal of Experimental Medicine*.

[109] Reddy K., Cusack C. L., Nnah I. C. (2016). Dysregulation of nutrient sensing and CLEARance in presenilin deficiency. *Cell Reports*.

[110] Wan O. W., Chung K. K. K. (2012). The role of alpha-synuclein oligomerization and aggregation in cellular and animal models of Parkinson’s disease. *PLoS One*.

[111] Sánchez-Danés A., Richaud-Patin Y., Carballo-Carbajal I. (2012). Disease-specific phenotypes in dopamine neurons from human iPS-based models of genetic and sporadic Parkinson’s disease. *EMBO Molecular Medicine*.

[112] Fernandes H. J. R., Hartfield E. M., Christian H. C. (2016). ER stress and autophagic perturbations lead to elevated extracellular α-synuclein in *GBA-N370S* Parkinson’s iPSC-derived dopamine neurons. *Stem Cell Reports*.

